# Analysis of the status of social frailty in Chinese older adults with cardiovascular and cerebrovascular diseases: a national cross-sectional study

**DOI:** 10.3389/fpubh.2023.1022208

**Published:** 2023-05-24

**Authors:** Xin Qi, Na Jia, Jiabin Hu, Ling-bing Meng, Ping Zeng, Junmeng Liu, Jing Shi, Xuezhai Zeng, Hui Li, Qiuxia Zhang, Juan Li, Deping Liu

**Affiliations:** ^1^Department of Cardiology, Beijing Hospital, National Center of Gerontology, Institute of Geriatric Medicine, Chinese Academy of Medical Sciences, Beijing, China; ^2^Health Service Department of the Guard Bureau of the Joint Staff Department, Beijing, China; ^3^The MOH Key Laboratory of Geriatrics, Beijing Hospital, National Center of Gerontology, Institute of Geriatric Medicine, Chinese Academy of Medical Sciences, Beijing, China; ^4^Department of Geriatrics, Beijing Hospital, National Center of Gerontology, Institute of Geriatric Medicine, Chinese Academy of Medical Sciences, Beijing, China; ^5^China Research Center on Ageing, Beijing, China; ^6^Institute of Psychology, Chinese Academy of Sciences, Beijing, China

**Keywords:** social frailty, cardiovascular and cerebrovascular disease, Chinese, healthy aging, older adults

## Abstract

**Background:**

Social frailty is one type of frailty. Physical frailty with cardiovascular and cerebrovascular diseases (CCVD) have been studied a lot, but less research on social frailty.

**Objectives:**

To study the prevalence, related risk factors and regional differences of social frailty with CCVD in Chinese older adults.

**Methods:**

SSAPUR was a national cross-sectional survey. Participants aged 60 years or older were recruited in August 2015. Demographic data and information regarding family, health and medical conditions, living environment conditions, social participation, spiritual and cultural life, and health condition were obtained. Social frailty was assessed in five areas (HALFE Social Frailty Index) including inability to help others, limited social participation, loneliness, financial difficulty, and living alone. The prevalence of CCVD with social frailty, related risk factors and regional differences in CCVD with social frailty were studied.

**Results:**

A total of 222,179 participants were enrolled. 28.4% of them had CCVD history. The prevalence of social frailty in the CCVD group was 16.03%. In CCVD participants, compared with the group without social frailty, there were significant differences in gender, age, urban–rural distribution, ethnicity, marital status, and education levels in the social frailty group. Significant differences were also found in physical exercise participation, health status, cataract, hypertension, diabetes mellitus, hospitalization within 1 year, self-assessed health status, crutch or wheelchair usage, urinary and fecal incontinence, need for care from others, fall history, housing satisfaction, and self-assessed happiness in the social frailty group. Women with CCVD had a higher prevalence of social frailty than men. By age in CCVD with social frailty, the highest prevalence was found in participants 75–79 years old. The prevalence of CCVD was significant difference between social frailty in urban and rural group. The prevalence of social frailty with CCVD was significantly different in different regions. The highest prevalence was 20.4% in southwest area, and the lowest prevalence was 12.5% in northeast with area.

**Conclusion:**

The prevalence of social frailty among the CCVD older adults is high. Factors such as gender, age, region, urban–rural residence, and the state of the disease may be associated with social frailty.

## Introduction

It is estimated that over 300 million Chinese people will be 60 years of age or older by 2025 (1). With this aging demographic, the prevalence of chronic diseases like cardiovascular and cerebrovascular diseases (CCVDs) and frailty will increase. CCVDs are a major cause of death among Chinese people, adding to the public health burden in China (2). Frailty describes a state of vulnerability due to an age-related decline in several physiological systems (3) and is associated with a considerably increased risk of falling, disability, hospitalization, and mortality (4, 5). Frailty has become a major public health concern in the current context of aging due to its impact on the older adults access’s to medical resources and age-related services. Frailty has received notable attention in the healthcare community in recent years due to its potentially adverse consequences for the older adults and society as a whole.

Existing research on risk factors of frailty mainly focuses on sociodemographic, physiological, and biological risk factors (6–11). In recent years, frailty has evolved from a concept focused on physical frailty to one that encompasses multiple health aspects (physical, psychological, and social) (12–21). Physical frailty indicates physical vulnerability, whereas social frailty is conceptualized as being at risk of losing or having lost sufficient social support, activities, or resources required to fulfill basic social needs (12, 15, 16, 20, 22–35). Several instruments have previously been used to assess social frailty among community-dwelling older adults, most commonly evaluating social activities, social support, social networks, loneliness, and living alone (12, 16, 20, 22, 25, 36–49). One study reported a nearly 30% overlap between physical and social frailty (24). Social frailty is a risk factor for long-term mortality and disability, and has a prevalence of approximately 18%–30% among community-dwelling older adults (24, 50–52). Research on social frailty is important, particularly in the context of situations such as quarantine or physical isolation, which have recently arisen due to the COVID-19 pandemic (32, 37, 53).

Frailty is believed to have a bidirectional effect on cardiovascular diseases (32, 33). Emerging evidence suggests that frailty is a risk factor for CCVDs, even after accounting for subclinical atherosclerosis (27). Conversely, CCVD risk factors and risk scores may predict frailty (33, 34): frailty is associated with a higher risk of CCVD prevalence (18). Therefore, frailty can be considered both a consequence of and a potential risk factor for CCVDs (18, 27, 33, 35, 37, 41, 42). Existing reports on the prevalence of frailty in the older adults with CCVDs are based on a unidimensional view of frailty in which only physical deficits are considered when determining frailty. The prevalence of social frailty in Chinese older adults with CCVDs has not been reported. Regional differences in the prevalence of social frailty may exist in the older adults in China. Therefore, the characteristics of the older adults with CCVDs combined with social frailty need to be investigated. This study uses data from the fourth Sample Survey of the Aged Population in Urban/Rural China (SSAPUR) conducted in 2015 and provides reference information for early interventions for CCVD patients with social frailty.

## Materials and methods

### Study population

Data were obtained from the database of the fourth SSAPUR, conducted by the China National Committee on Ageing in 2015. Chinese citizens aged 60 or above were surveyed to compile the largest database of older people in China. The sampling method of the survey was introduced in a previous study (43). The SSAPUR covered 31 provinces, autonomous regions, municipalities, and the Xinjiang Production and Construction Corps, including 466 counties (districts), 1,864 townships (sub-districts), and 7,456 village (residential) committees. The questionnaire covered nine domains: demographic information, family situation, health status, healthcare and nursing services, economic status, social activity, living environment, and spiritual and cultural life (including psychological status), and was divided into simplified and detailed forms, as introduced in a previous study (43). CCVDs included coronary heart disease, angina pectoris, and stroke as self-reported by the participants.

The research protocol was approved by the National Bureau of Statistics (No. [2014] 87) and the Ethics Committee of the Beijing Hospital (2021BJYYEC-294-01). All participants provided written informed consent before completing the questionnaire. The number of collected samples was 2,24,142.

### Definition of social frailty

#### Social frailty screening questionnaire: HALFE scale

To identify and assess social frailty, we considered five categories based on previous studies (7, 12–16, 21, 22, 44–50): inability to help others, limited social participation, loneliness, financial difficulty, and living alone. “HALFE” is an acronym for these five components: Help, pArticipation, Loneliness, Financial situation, and living alonE. The ability to help others was measured by asking participants if they were able to help their friends or family within the past 12 months. If participants responded “no,” the item was scored 1. Limited social participation was assessed by asking participants if they had engaged in any social or leisure activities in the past 12 months. If participants responded “no,” the item was scored 1. Loneliness was scored 1 if participants responded “yes” to feeling lonely. The financial situation was divided into five grades: very wealthy, relatively wealthy, basically enough, relatively difficult, or very difficult. Financial difficulty was scored 1 if participants had a “relatively difficult” or “very difficult” financial situation. Living alone was scored 1 if the participants lived alone. The total score on the HALFE scale ranges from 0 to 5, with a total score ≥ 3 indicating social frailty.

### Statistical analysis

The characteristics of subjects with and without social frailty were compared using one-way analysis of variance (ANOVA) for normal-distributed quantitative data and the Chi-square test for categorical data. Multivariate logistic regression was performed to estimate the adjusted odds ratios and 95% confidence intervals (CIs) of variables associated with social frailty. *p*-value < 0.05 was chosen as the threshold for statistical significance. All statistical analyses were performed using SPSS 24.0 (IBM Corp., Armonk, NY, United States).

## Results

### Prevalence of social frailty in CCVD group and non-CCVD group

**Figure 1:**
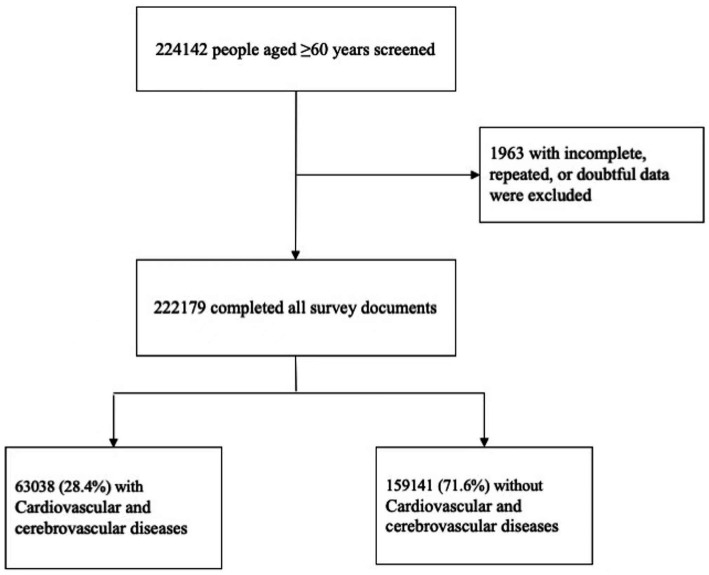
Study flowchart.

Data of 2,24,142 participants were collected from SSAPUR 2015. After excluding 1,963 cases with missing, doubtful, or duplicate data, 2,22,179 cases were included in the final analysis. Among the participants, 63,038 (28.4%) had a history of CCVDs and 1,59,141 (71.6%) did not have CCVDs (Figure 1).

The prevalence of social frailty was 16.03% in the CCVD group and 14.9% in the non-CCVD group, with significant differences between the two groups (*p* < 0.001). There were significant differences in gender, age, urban–rural distribution, ethnicity, marital status, and education levels in the CCVD group with social frailty than without social frailty (Table 1). Women with CCVDs had a higher prevalence of social frailty than men with CCVDs (Table 1).

**Table 1:** The prevalence of social frailty status based on demographic information in the group with and without CCVD.

		Non-CCVD					CCVD				
		Non-SF *n* (%)	SF *n* (%)	Total	χ^2^	*P*-value	Non-SF *n* (%)	SF n (%)	Total	χ^2^	*P*-value
Gender	Female	68,501 (85.4)	11,740 (14.6)	80,241	7.108	0.008	29,891 (83.5)	5,906 (16.5)	35,797	12.692	<0.001
	Male	66,981 (84.9)	11,919 (15.1)	78,900			23,033 (84.6)	4,208 (15.4)	27,241		
Ethnicity	Han ethnic group	127,156 (85.4)	21,652 (14.6)	148,808	181.274	<0.001	50,182 (84.1)	9,512 (15.9)	59,694	10.052	0.002
	Non-Han ethnic group	8,326 (80.6)	2007 (19.4)	10,333			2,742 (82.0)	602(18.0)	3,344		
Educational levels	Non-Illiteracy	97,973 (86.9)	14,786 (13.1)	112,759	940.187	<0.001	37,746 (85.5)	6,400 (14.5)	44,146	261.693	<0.001
	Illiteracy	37,509 (80.9)	8,873 (19.1)	46,382			15,178 (80.3)	3,714 (19.7)	18,892		
Marriage status	Spousal presence	106,132 (90.9)	10,605 (9.1)	116,737	11573.141	<0.001	39,572 (89.2)	4,808 (10.8)	44,380	3022.222	<0.001
	Single	29,350 (69.2)	13,054 (30.8)	42,404			13,352 (71.6)	5,306 (28.4)	18,658		
Age	60–64	49,270 (87.5)	7,011 (12.5)	56,281	588.488	<0.001	14,229 (84.4)	2,627 (15.6)	16,856	48.623	<0.001
	65–69	32,286 (85.5)	5,470 (14.5)	37,756			12,363 (83.9)	2,366 (16.1)	14,729		
	70–74	21,029 (83.3)	4,231 (16.7)	25,260			9,712 (82.9)	2009 (17.1)	11,721		
	75–79	15,841 (81.7)	3,541 (18.3)	19,382			7,981 (82.7)	1,671 (17.3)	9,652		
	80–84	10,352 (82.3)	2,221 (17.7)	12,573			5,583 (85.4)	953 (14.6)	6,536		
	85 and over	6,704 (85.0)	1,185 (15.0)	7,889			3,056 (86.2)	488 (13.8)	3,544		
Urban/ rural	Urban	72,454 (89.5)	8,506 (10.5)	80,960	2475.505	<0.001	30,613 (88.2)	4,087 (11.8)	34,700	1042.973	<0.001
	Rural	83,028 (80.6)	15,153 (19.4)	78,181			22,311 (78.7)	6,027 (21.3)	28,338		

CCVD, cardiovascular and cerebrovascular diseases; SF, social frailty.

Significant differences were also found in physical exercise participation, health status, the prevalence of cataract, hypertension, and diabetes mellitus, hospitalization within 1 year, self-assessed health status, crutch or wheelchair usage, urinary and fecal incontinence, need for care from others, fall history, housing satisfaction, and self-assessed happiness in the CCVD group with social frailty (Table 2). However, no significant differences were found in cataract and hypertension prevalence in the non-CCVD group with social frailty compared with the non-CCVD participants without social frailty (Table 2).

**Table 2:** The prevalence of social frailty status based on health/medical conditions and social participation in the group with and without CCVD.

		Non-CCVD					CCVD				
		Non-SF *n* (%)	SF *n* (%)	Total	χ^2^	*P*-value	Non-SF *n* (%)	SF n (%)	Total	χ^2^	*P*-value
Physical exercise	Once a week or more	64,133 (87.8)	8,886 (12.2)	73,019	775.593	<0.001	24,899 (85.9)	4,103 (14.1)	29,002	143.500	<0.001
	No	71,349 (82.2)	14,773 (17.2)	86,122			28,025 (82.3)	6,011 (17.7)	34,036		
Cataract/ glaucoma	No	114,566 (85.2)	19,931(14.8)	134,497	0.526	0.468	44,893 (84.3)	8,373 (15.7)	53,266	63.240	<0.001
	Yes	20,916 (84.9)	3,728 (15.1)	24,644			8,031 (82.2)	1741 (17.8)	9,772		
Hypertension	No	92,897 (85.1)	16,308 (14.9)	109,205	1.223	0.269	21,784 (82.0)	4,769 (18.0)	26,553	125.0.034	<0.001
	Yes	42,585(85.3)	7,351 (14.7)	49,936			31,140 (85.4)	5,345 (14.6)	36,485		
Diabetes mellitus	No	125,578 (80.6)	22,182 (19.4)	147,760	34.556	<0.001	39,820 (82.0)	8,736 (18.0)	48,556	594.997	<0.001
	Yes	9,904 (87.0)	1,477 (13.0)	11,381			13,104 (90.5)	1,378 (9.5)	14,482		
Osteopathy	No	80,719 (87.8)	11,183 (12.2)	91,902	1251.268	<0.001	31,751 (87.3)	4,617 (12.7)	36,368	715.773	<0.001
	Yes	54,763 (81.4)	12,476 (18.6)	67,239			21,173 (79.4)	5,497 (20.6)	26,670		
Cancer	No	134,023 (85.2)	23,307 (14.8)	157,330	30.229	<0.001	52,417 (84.0)	9,967 (16.0)	62,384	20.302	<0.001
	Yes	1,459 (80.6)	352 (19.4)	1811			507 (77.5)	147 (22.5)	654		
Lung diseases	No	124,464 (85.8)	20,612 (14.2)	145,076	563.182	<0.001	46,823 (84.9)	8,327 (15.1)	55,150	292.468	<0.001
	Yes	11,018 (78.3)	3,047 (21.7)	14,065			6,101 (77.3)	1787 (22.7)	7,888		
Dentures	No	101,075 (85.0)	17,803 (15.0)	118,878	4.424	0.035	39,671 (84.4)	7,318 (15.6)	46,989	30.323	<0.001
	Yes	34,407 (85.5)	5,856 (14.5)	40,263			13,253 (82.6)	2,796 (17.4)	16,049		
Crutching using	No	124,022 (85.4)	21,244 (14.6)	145,266	77.404	<0.001	48,584 (84.2)	9,090 (15.8)	57,674	40.382	<0.001
	Yes	11,460 (82.6)	2,415 (17.4)	13,875			4,340 (80.9)	1,024 (19.1)	5,364		
Wheel chairs using	No	132,991 (85.1)	23,303 (14.9)	156,294	12.782	<0.001	51,889 (83.9)	9,954 (16.1)	61,843	6.375	0.012
	Yes	2,491 (87.5)	356 (12.5)	2,847			1,035 (86.6)	160 (13.4)	1,195		
Hospitalization within 1 year	No	106,361 (86.1)	17,224 (13.9)	123,585	377.772	<0.001	31,871 (85.5)	5,400 (14.5)	37,271	163.853	<0.001
	Once or more	29,121 (81.9)	6,435 (18.1)	35,556			21,053 (81.7)	4,714 (18.3)	25,767		
Self-awareness of healthy	Healthy	123,738 (86.6)	19,157 (13.4)	142,895	2358.570	<0.001	40,721 (86.3)	6,443 (13.7)	47,164	789.891	<0.001
	Unhealthy	11,744 (72.3)	4,502 (27.7)	16,246			12,203 (76.9)	3,671 (23.1)	15,874		
Fecal incontinence	No	128,018 (85.2)	22,223 (14.8)	1,150,241	11.979	0.001	41,820 (82.7)	8,748 (17.3)	50,568	2,980,991	<0.001
	Yes	7,464 (83.9)	1,436 (16.1)	8,900			11,104 (89.0)	1,366 (11.0)	12,470		
Urinary incontinence	No	125,671 (85.4)	21,525 (14.6)	147,196	91.743	<0.001	39,822 (82.9)	8,221 (17.1)	48,043	170.852	<0.001
	Yes	9,811 (82.1)	2,134 (17.9)	11,945			13,102 (87.4)	1893 (12.6)	14,995		
Supporting supplies Hearing aids	No	133,481 (85.1)	23,331 (14.9)	156,812	1.146	0.284	52,079 (84.0)	9,946 (16.0)	62,025	0.223	0.637
	Yes	2001 (85.9)	328 (14.1)	2,329			845 (83.4)	168 (16.6)	1,013		
Diapers	No	134,236 (85.1)	23,441 (14.9)	157,677	0.001	0.979	52,431 (84.0)	10,009 (16.0)	62,440	1.028	0.311
	Yes	1,246 (85.1)	218 (14.9)	1,464			493 (82.4)	105 (17.6)	598		
Need care from others	No	116,947 (85.5)	19,801 (14.5)	136,748	114.864	<0.001	45,046 (84.3)	8,405 (15.7)	53,451	26.653	<0.001
	Yes	18,535 (82.8)	3,858 (17.2)	22,393			7,878 (82.2)	1709 (17.8)	9,587		
Number of chronic diseases	Less than 2	95,184 (86.9)	14,339 (13.1)	109,523	873.913	<0.001	8,771 (86.2)	1,409 (13.8)	10,180	43.759	<0.001
	2 or more	40,298 (81.2)	9,320 (18.8)	49,618			44,153 (83.5)	8,705 (16.5)	52,858		
Falls	No	117,686 (86.4)	18,482 (13.6)	136,168	1247.462	<0.001	41,231 (85.8)	6,799 (14.2)	48,030	534.171	<0.001
	Yes	17,796 (77.5)	5,177 (22.5)	22,973			11,693 (77.9)	3,315 (22.1)	15,008		
Housing satisfaction	Satisfied	121,007 (87.2)	17,745 (12.8)	138,752	3693.782	<0.001	45,552 (86.0)	7,408 (14.0)	52,960	1039.944	<0.001
	Dissatisfied	14,475 (71.0)	5,914 (29.0)	20,389			7,372 (73.1)	2,706 (26.9)	10,078		
Happiness	Happy	127,448 (85.6)	21,517 (14.4)	148,965	328.350	<0.001	49,733 (84.4)	9,215 (15.6)	58,948	114.418	<0.001
	Unhappy	8,034 (79.0)	2,142 (21.0)	10,176			3,191 (78.0)	899 (22.0)	4,090		
Self-care ability	Fully independent	117,378 (86.2)	18,768 (13.8)	136,146	870.731	<0.001	38,998 (85.0)	6,864 (15.0)	45,862	145.117	<0.001
	Dependent	18,104 (78.7)	4,891 (21.3)	22,995			13,926 (81.1)	3,250 (18.9)	17,176		

CCVD, cardiovascular and cerebrovascular diseases; SF, social frailty.

### Sub-group analysis of the prevalence of social frailty in CCVD patients

**Figure 2:**
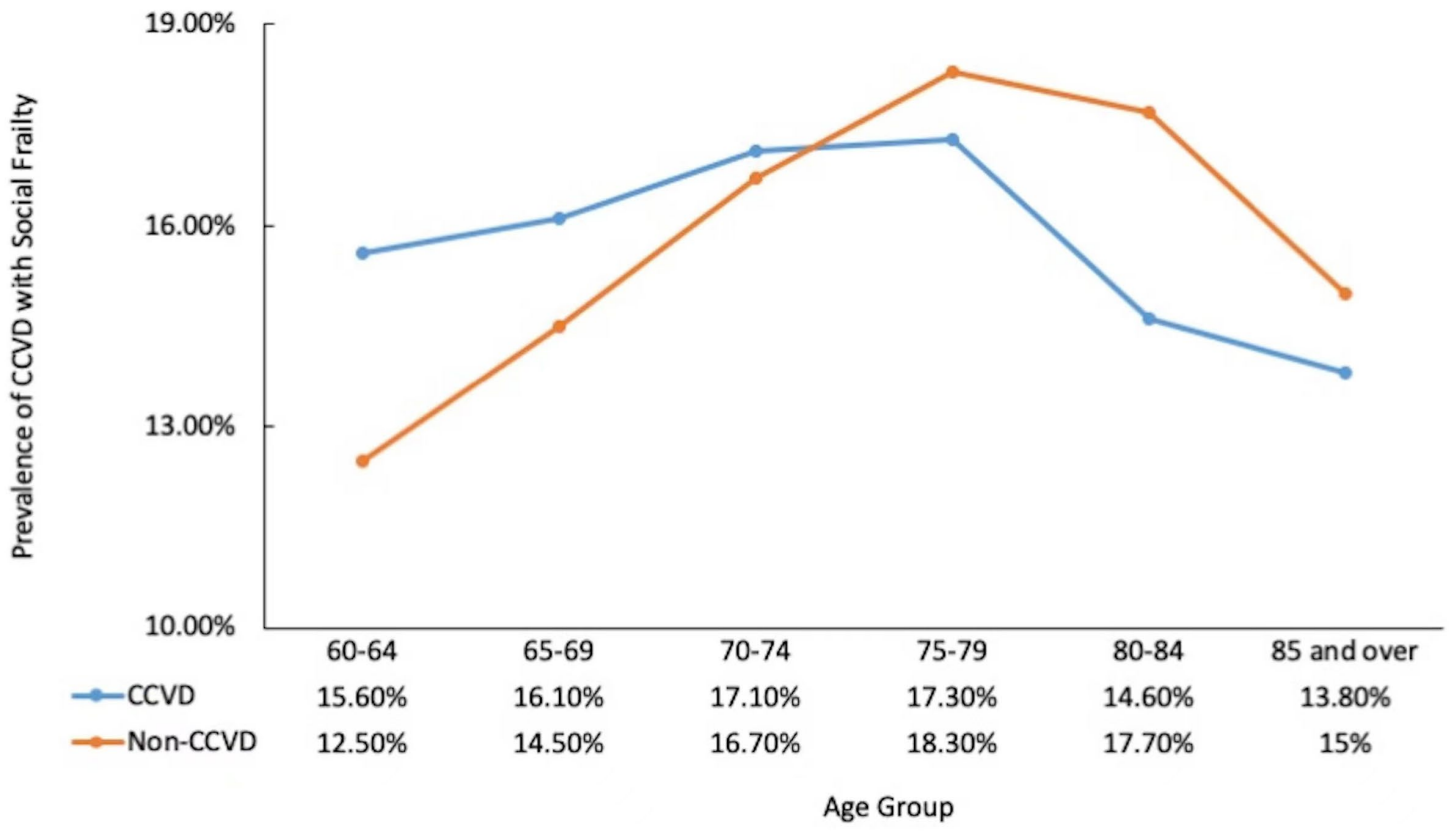
Prevalence of social frailty by age and CCVD status CCVD, cardiovascular and cerebrovascular diseases.

The highest prevalence of social frailty in the CCVD group was observed in participants aged 75–79. For participants aged below 75, the prevalence of social frailty was higher in the CCVD group than in the non-CCVD group, and the prevalence rate increased with age. The opposite trend was observed in participants aged above 75, with less prevalence of social frailty in the CCVD group than in the non-CCVD group, and the prevalence rate decreased with age (see Table 3; Figure 2 for details).

**Table 3:** The prevalence of social frailty in the group with and without CCVD by ages.

	Non-CCVD		CCVD	
Age group	Prevalence of SF	CI (%)	Prevalence of SF	CI (%)
60–64	12.5%	12.19–12.74	15.6%	15.04–16.14
65–69	14.5%	14.14–14.85	16.1%	15.48–16.66
70–74	16.7%	16.29–17.22	17.1%	16.47–17.83
75–79	18.3%	17.73–18.82	17.3%	16.57–18.08
80–84	17.7%	17.00–18.34	14.6%	13.75–15.46
85 and over	15.0%	14.25–15.83	13.8%	12.67–14.94

CCVD, cardiovascular and cerebrovascular diseases.

**Figure 3:**
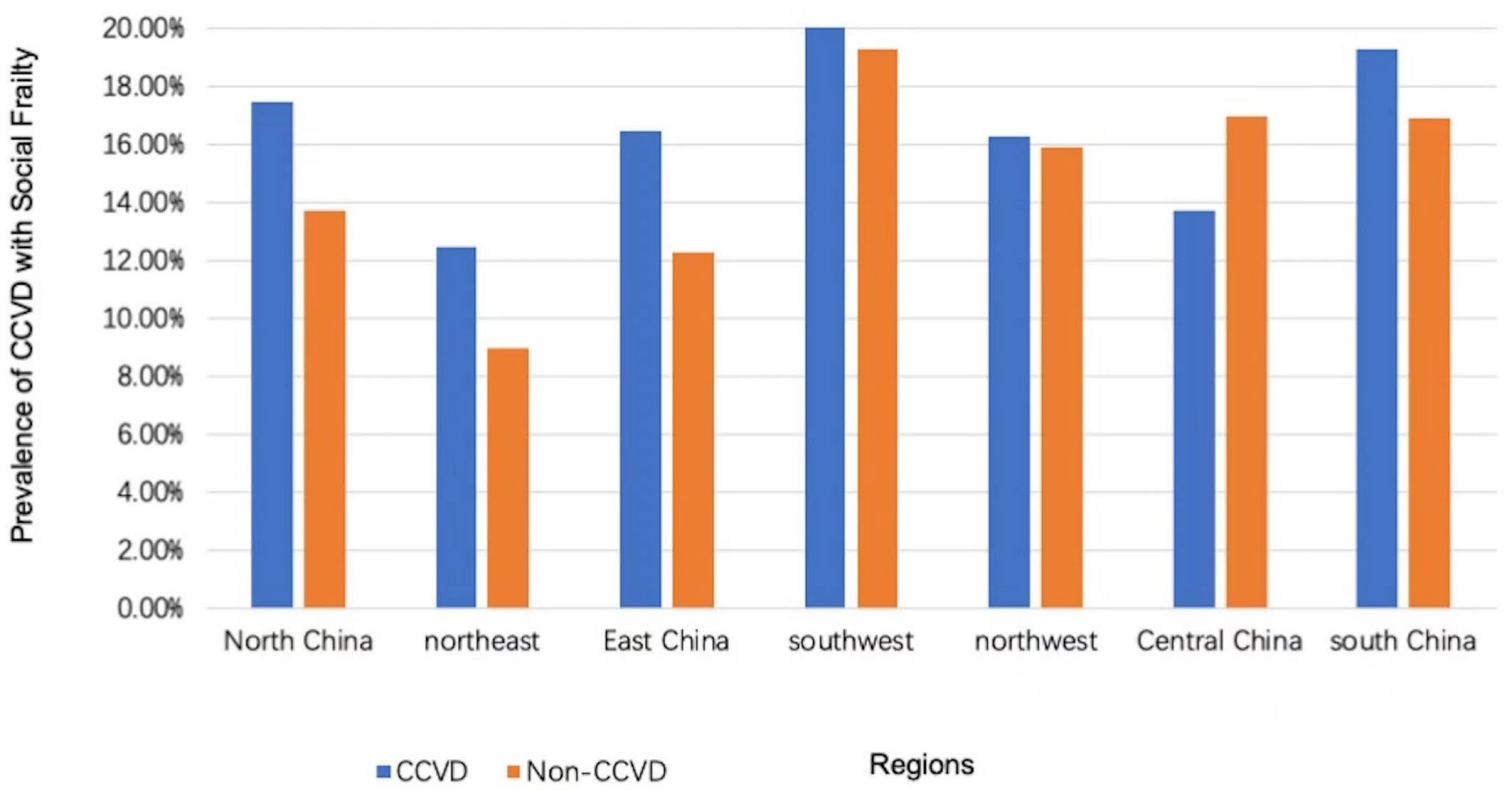
Prevalence of social frailty in the group with and without CCVD in different regions of China CCVD, cardiovascular and cerebrovascular diseases.

**Figure 4:**
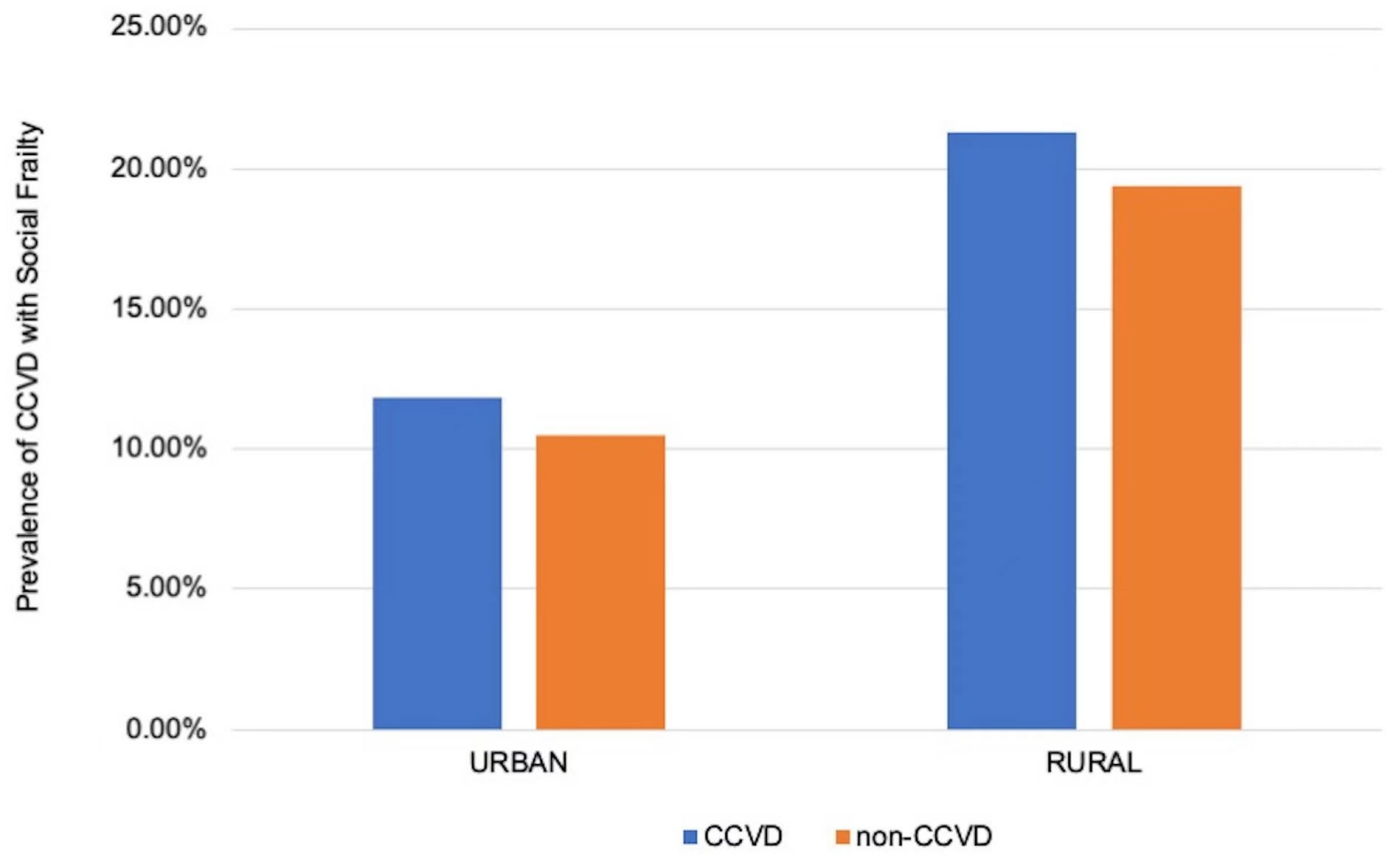
Prevalence of social frailty in the group with and without CCVD in urban and rural area of China CCVD, cardiovascular and cerebrovascular diseases.

There existed significant differences in the prevalence of social frailty in CCVD patients with hypertension and CCVD patients with diabetes. The prevalence of social frailty in CCVD patients also varied based on region (*p* < 0.001, see Table 4; Figure 3 for details): Southwest China had the highest prevalence of social frailty (20.4%) and the Northeast had the lowest prevalence rate (12.5%). The prevalence of social frailty combined with CCVDs was 11.8% in urban areas and 21.3% in rural areas, with significant differences (see Figure 4).

**Table 4:** The prevalence of social frailty in different regions in the group with and without CCVD.

	Non-CCVD					CCVD				
	Non-SF *n* (%)	SF *n* (%)	Total	χ^2^	*P*-value	Non-SF *n* (%)	SF *n* (%)	Total	χ^2^	*P*-value
North China	13,640(86.3)	2,171(13.7)	15,811	1175.768	0.000	7,383(82.5)	1,562(17.5)	8,945	275.231	0.000
Northeast China	10,123(91.0)	1,003(9.0)	11,126			6,521(87.5)	929(12.5)	7,450		
East China	47,436(87.7)	6,678(12.3)	54,114			13,424(83.5)	2,651(16.5)	16,075		
Southwest China	23,112(80.7)	5,518(19.3)	28,630			5,778(79.6)	1,478(20.4)	7,256		
Northwest China	7,445(84.1)	1,407(15.9)	8,852			3,816(83.7)	742(16.3)	4,558		
Central China	17,236(83.0)	3,537(17.0)	20,773			13,436(86.3)	2,173(13.7)	15,573		
South China	16,490(83.1)	3,345(16.9)	19,835			2,566(80.7)	615(19.3)	3,181		

CCVD, cardiovascular and cerebrovascular diseases.

Multivariate regression analysis showed that gender, age, educational level, marital status, living in an urban vs. rural environment, physical exercise, number of comorbid chronic diseases, lung diseases, cancer, wheelchair usage, fecal incontinence, fall history, housing satisfaction, self-assessed happiness, and self-care ability were all associated with social frailty in CCVD patients (see Table 5).

**Table 5:** Factors associated with social frailty in the group with CCVD.

Variables	Comparison	Groups	Chi-square value	*P-*value	OR	95%CI
Gender	Female	Male	139.871	<0.001	1.356	1.290–1.427
Educational levels	Illiteracy	Non-Illiteracy	10.691	0.001	1.094	1.037–1.115
Marriage status	Single	Spousal presence	3195.182	<0.001	4.389	4.170–4.620
Age	85 and over	60–64	304.836	<0.001	0.349	0.310–0.393
		65–69	261.724	<0.001	0.378	0.337–0.426
		70–74	215.989	<0.001	0.416	0.370–0.467
		75–79	133.836	<0.001	0.502	0.446–0.564
		80–84	24.167	<0.001	0.733	0.647–0.830
Urban or rural area	Rural	Urban	575.700	<0.001	1.815	1.729–1.906
Physical exercise	Once a week or more	No	4.341	0.037	0.949	0.903–0.997
Number of chronic diseases	2 or more	Less than 2	6.363	0.012	1.114	1.024–1.212
Wheel chairs using	Yes	No	4.729	0.030	0.822	0.688–0.981
Housing satisfaction	Dissatisfied	Satisfied	733.424	<0.001	2.126	2.013–2.245
Cataract/ glaucoma	Yes	No	9.300	0.002	1.100	1.035–1.169
Hypertension	Yes	No	18.498	<0.001	0.889	0.843–0.938
Diabetes mellitus	Yes	No	171.904	<0.001	0.626	0.583–0.671
Osteopathy	Yes	No	169.766	<0.001	1.430	1.355–1.509
Lung diseases	Yes	No	67.305	<0.001	1.306	1.226–1.392
Cancer	Yes	No	22.781	<0.001	1.624	1.331–1.982
Dentures	Yes	No	6.180	0.013	1.068	1.014–1.124
Fecal incontinence	Yes	No	120.794	<0.001	0.676	0.630–0.724
Falls	Yes	No	189.009	<0.001	1.435	1.363–1.510
Happiness	Unhappy	Happy	38.299	<0.001	1.303	1.199–1.418
Self-care ability	Dependent	Fully independent	12.624	<0.001	1.103	1.045–1.164

CCVD, cardiovascular and cerebrovascular diseases; SF, social frailty.

## Discussion

To the best of our knowledge, this study conducts the most extensive survey on social frailty among the older adults in urban and rural China to date. The study investigated the prevalence of social frailty in the older adults with CCVDs in China and identified health risk factors and socioeconomic factors associated with social frailty.

The findings showed that 16.03% of the older adults with CCVDs also suffered from social frailty. This confirms that CCVDs and frailty are common among community-dwelling older adults. Age is a significant predictor of frailty. Thinuan et al. showed that age was associated with a higher prevalence of prefrailty and frailty. Frailty, in turn, is associated with an increased occurrence of CCVDs (32, 33, 35). This study showed that the prevalence of social frailty combined with CCVDs varied with age: social frailty increased with age till 80 years, after which it decreased with age. The highest prevalence of social frailty was observed in participants with CCVDs aged 75–79 years, which may be because many older adults with CCVDs and social frailty do not survive to be 80. Social frailty is associated with lower dietary intake, poor diet quality, and poor nutrition among community-dwelling older men, which in turn are associated with physical frailty, cognitive decline, hospitalization, and mortality (20–26). The prevalence of social frailty decreased in those with CCVDs aged 80 and beyond, possibly because older adults require more companionship, are less likely to live alone, and have more social interactions, reducing the prevalence of social frailty.

Regarding gender, in the case of the CCVD group, the prevalence of social frailty was higher in women than in men, whereas, for non-CCVD participants, the prevalence of social frailty was higher in men than in women. The prevalence of social frailty in patients with heart failure can be as high as 66.5% (33). Some studies have shown that the prevalence of heart failure is higher in older adults women than in older adults men: heart failure prevalence is 10 per 1,000 people after the age of 65 years, with 8.6% of men and 11.5% of women aged > 80 years afflicted with heart failure (32, 33). Men diagnosed with frailty syndrome are more likely to suffer from heart failure than women (35). Thus, the relationship between gender and social frailty needs further study.

Furthermore, the survey showed a significant difference in the prevalence of social frailty combined with CCVDs across different regions in mainland China. According to the China Cardiovascular Health Report 2016, the prevalence of CCVDs is significantly higher in the north than in the south, with the highest prevalence rate in Northeast China, followed by North China, and a lower prevalence rate in South China, Central China, East China, Northwest China, and Southwest China. As for the prevalence of social frailty in the older adults with CCVDs, Southwest China had the highest prevalence rate of 20.4%, and the Northeast had the lowest prevalence rate of 12.5%. These differences may be related to cultural factors and social economies of the regions and need further exploration.

The findings also showed a significant difference in the prevalence of social frailty among the older adults with CCVDs between urban and rural areas in mainland China, with a significantly higher prevalence in rural people than in urban people. This difference may be related to the overall higher economic and cultural levels and living conditions associated with urban environments. Therefore, eliminating the urban–rural gap and economic development are critical to alleviating social frailty.

An interesting phenomenon is the occurrence of social frailty in hypertensive older adults. Hypertension is one of the most important risk factors for CCVDs and its prevalence increases with age, affecting endothelial function and leading to oxidative stress, inflammation, and atherosclerosis. For non-CCVD older adults, the prevalence of social frailty in those with and without hypertension was 14.7% and 14.9%, respectively, without significant differences. For the older adults with CCVDs, the prevalence of social frailty in those with and without hypertension was 14.6% and 18.0%, respectively, with a significant difference. This may be related to the long medical history of patients with hypertension, and their increased awareness of the need for long-term medication, follow-up, monitoring, and exercise.

Hyperglycemia is one of the most common comorbidities in older adults, driving inflammation and oxidative stress, leading to endothelial dysfunction, with a negative impact on frail patients. Hyperglycemia is frequently observed in frail older adults and is an independent predictor of poor health outcomes. High glucose levels may increase the risk of frailty in older adults, causing physical impairment in frail hypertensive older adults (5, 39, 54). However, in this study, for the non-CCVD group, the prevalence of social frailty with and without diabetes was 13% and 19.4%, respectively, with significant differences. For the older adults with CCVDs, the prevalence of social frailty with and without diabetes was 9.5% and 18.0%, respectively. The lower prevalence of social frailty among diabetes patients may be because treatment for diabetes requires keeping a close watch over one’s blood sugar levels with a combination of medications, exercise, and diet. Patients with diabetes go to the hospital every few months to see their doctors, talk with other diabetes patients, and often participate in sports activities, which increases their social participation, thus lowering the risk of social frailty.

The findings of this study have important implications for clinical practice. More research is required on social frailty as it influences health outcomes through health behaviors and lifestyles. Social frailty cannot be screened or treated simply with medications but requires comprehensive attention to the social environment; thus, it is necessary to pay attention to social frailty in older adults and actively manage consequent problems and implement relevant interventions (32, 33, 35, 55).

The study also has some limitations. Firstly, the data on CCVDs were self-reported and might be subject to memory bias. Secondly, owing to the cross-sectional design of the study, causality could not be explored. This should be investigated in a future prospective study. Thirdly, the categories of social frailty were based on recent studies rather than an established method. Therefore, future research on instrument development to measure social frailty is needed.

## Conclusion

This study shows that there is a high prevalence of social frailty among the older adults with CCVDs in China. Factors such as gender, age, region, urban–rural residence, and the state of the disease influence the prevalence of social frailty. Future research should further study the relevant factors affecting the occurrence and development of social frailty and its relationship with physical frailty to improve frailty prognosis and the quality of life of older patients with CCVDs.

## Data availability statement

The raw data supporting the conclusions of this article will be made available by the authors, without undue reservation.

## Author contributions

XQ and NJ wrote the various drafts of the manuscript. XQ, LM, and PZ conducted the statistical analyses, with advice from JH, JmL, JS, XZ, HL and JuL were involved in data interpretation. DL, QZ, JuL, and XQ conceived and designed this study. Drafts of the manuscript were revised for important scientifific content by XQ, NJ, JH, LM, PZ, JmL, JS, XZ, HL, QZ, JuL and DL. DL is the guarantor of this work and, as such, had full access to all the data in the study and takes responsibility for the integrity of the data and the accuracy of the data analysis. All authors gave final approval of the version to be published.

## Funding

The present study was funded by the National Key R&D Program of China (grant nos. 2020YFC2003000 and 2020YFC2003001). DL has full access to all study data and has final responsibility for the decision to submit for publication.

## Conflict of interest

The authors declare that the research was conducted in the absence of any commercial or financial relationships that could be construed as a potential conflict of interest.

## Publisher’s note

All claims expressed in this article are solely those of the authors and do not necessarily represent those of their affiliated organizations, or those of the publisher, the editors and the reviewers. Any product that may be evaluated in this article, or claim that may be made by its manufacturer, is not guaranteed or endorsed by the publisher.
